# User-centred participatory design of visual cues for isolation precautions

**DOI:** 10.1186/s13756-019-0629-9

**Published:** 2019-11-19

**Authors:** Lauren Clack, Manuel Stühlinger, Marie-Theres Meier, Aline Wolfensberger, Hugo Sax

**Affiliations:** 1 Division of Infectious Diseases and Hospital Epidemiology, University Hospital Zurich and University of Zurich, Raemistrasse 100 / HAL14, 8091 Zurich, Switzerland; 2 Department of Management, Technology and Economics, ETH Zurich, Zurich, Switzerland

**Keywords:** Infection control, Isolation precautions, Personal protective equipment, Human factors, User-centred design, Participatory design, Co-design, Visual cues, Signage

## Abstract

**Background:**

Isolation precautions are intended to prevent transmission of infectious agents, yet healthcare provider (HCP) adherence remains suboptimal. This may be due to ambiguity regarding the required precautions or to cognitive overload of HCPs. In response to the challenge of changing HCP behaviour, increasing attention should be paid to the role of engineering controls and facility design that incorporate human factors elements. In the current study, we aimed to develop an isolation precaution signage system that provides visual cues, serves as a cognitive aid at the point of care, and removes ambiguity regarding which precautions are necessary (e.g. masks, gowns, gloves, single rooms) when caring for isolated patients.

**Methods:**

We employed a user-centred, participatory design approach in which HCPs were actively involved in generating an *isolation precaution signage system* based on human factors design principles*.* HCPs were purposefully sampled for each design phase to include a representative sample of potential system users. We conducted front-end analysis through interviews and observations to identify challenges related to the existing signage and to establish design requirements for new signage. This was followed by the creation of user personas, design thinking workshops, and prototyping, which then underwent iterative cycles of evaluation. Graphical symbols were developed and tested for comprehensibility.

**Results:**

Front-end analysis revealed several barriers to use of the current signage system such as unclear target audience, low signal-to-noise ratio, and ambiguity regarding the applicable precautions. A comprehensive list of design requirements was generated. The project ultimately resulted in a collection of validated, comprehensible symbols and signs for contact, droplet, and airborne isolation, as well as the identification of several systems-level solutions for work re-organisation to improve compliance with isolation precautions.

**Conclusions:**

The introduction of visual cues in the form of signage offers a promising opportunity to make guidelines available directly at the frontline. Anecdotal evidence based on observations and interviews with HCP have shown that the current solution is superior to previous isolation signage. User-centred participatory design was a useful approach that holds potential for further improving design in healthcare settings.

## Introduction

Healthcare-associated infections (HAI) are a major threat to patient safety on a global level, even in high-income countries [1]. Adherence to recommended infection control practices, such as outlined by the Centers for Disease Control (CDC) recommendations on isolation precautions, decreases transmission of infectious agents in healthcare settings, significantly reducing the burden of HAI [2]. Transmission-based precautions are designed to prevent contact, droplet, or airborne transmission of infectious agents from a source to a susceptible host. In spite of the demonstrated effectiveness of such measures to prevent transmission, healthcare provider (HCP) compliance has been shown by observational studies to be suboptimal, with adherence to isolation and universal precautions ranging from 43 to 89%, depending on the care practice assessed [3–5].

Most attempts to improve adherence to standard precautions up to this point have focused on educating HCPs about the rationale and application of transmission-based precautions. While such initiatives have resulted in improved knowledge and attitudes, they usually lack an accompanying sustained change in behaviour [6, 7]. It has been suggested that HCP compliance with guidelines may be suboptimal due to high levels of ambiguity surrounding tasks (e.g. not knowing which guidelines are applicable) and expectations (e.g. not knowing what practice is acceptable or feasible) [8] and high cognitive load of HCPs [9]. In response to the challenge of changing behaviour, increasing attention is paid to the role of environmental restructuring approaches that incorporate human factors design elements to improve adherence [10]. One such opportunity for incorporation of human factors principles is the design of signage, indicating which precautions should be used and when, at the place where their use is indicated.

Signage in healthcare, like in other settings, is used for communication with people with different languages, ages, and physiological challenges [11]. In healthcare specifically, signage should also communicate effectively with different categories of HCPs, who in the case of isolation precautions may require different information, and even with patients and their visitors [12]. Such signage should act as cognitive aids in the form of visual cues [13], and should remove ambiguity surrounding which precautions are necessary when treating isolated patients. Thus, these and several human factors considerations should be taken into account when designing and implementing such signage.

Participatory design, a user-centred design methodology in which end-users are actively involved in the design process, creates the opportunity to benefit from the experience and expertise of key stakeholders [14]. Participatory design methodology is well suited for use in healthcare, as it establishes a collaboration with frontline HCPs to develop practical solutions that correspond with frontline needs [15]. This project utilised human factors engineering principles and a user-centred participatory design approach to design a signage system that 1) provides visual cues and 2) removes ambiguity for HCPs about which precautions should be taken when treating isolated patients.

## Methods

### Study design

**Fig. 1:**
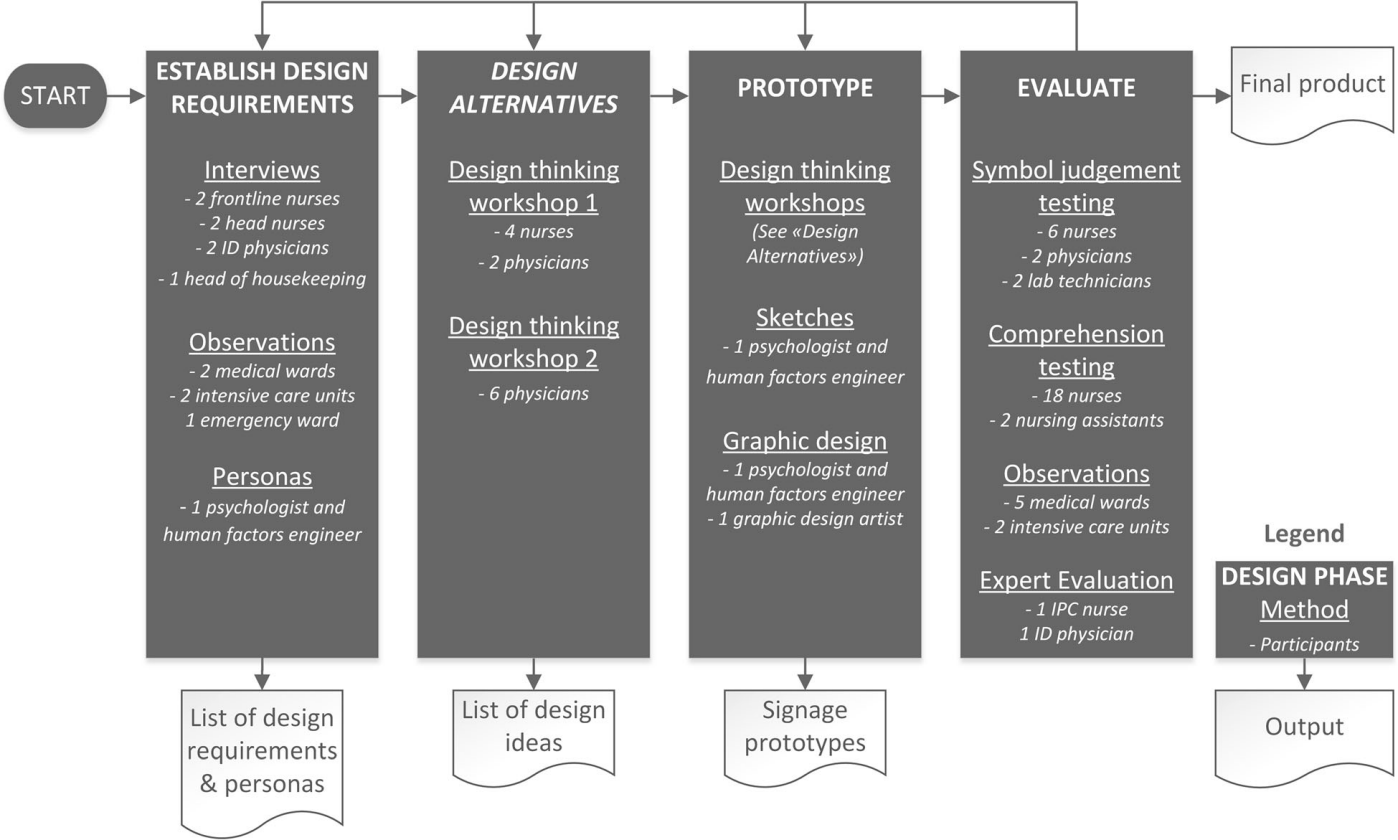
User-centred design process. Legend: Multiple methods were employed for each phase of the user-centred design process. Participants were purposefully recruited for each study phase to include a broad range of potential users of the system. ID, infectious diseases; IPC, infection prevention and control

The human factors-informed, user-centred, participatory design process employed in this project was composed of four stages, each resulting in an output that informed the subsequent activities throughout the iterative design lifecycle shown in Fig. 1 and described in the following sections [16]. The participatory process was facilitated by a psychologist with training in human factors engineering and extensive experience in the field of infection prevention and control (LC). An infection control nurse (MM) and two infectious diseases physicians (AW, HS) provided expert input throughout the process. These four individuals composed the design team.

### Study setting and participants

This project was carried out at the University Hospital of Zurich (USZ), a 900-bed, university-affiliated tertiary care hospital in 2014–2016. It was initiated in response to low reported adherence to isolation precautions and numerous critical incident reports related to isolation precautions. The project aimed to introduce signage for universal use throughout the hospital. Successful implementation of the new signage would require its use by several professional categories of HCPs (e.g. nurses, physicians, allied-care professionals, assistants) and hospital staff (e.g. housekeeping) working in several hospital settings (e.g. intensive care, emergency, general wards). Participants for each phase of the design process were thus purposefully sampled by professional category and by work setting in order to include a representative sample of potential users of the system (Fig. 1).

### Establishing design requirements – interviews and observations

Two primary methods were used to establish design requirements that would inform the subsequent signage design: 1) interviews with front-end users and 2) direct observations. We conducted semi-structured interviews with anticipated typical users of the isolation signage system and explored how isolation precautions are generally handled (*Could you walk me through the process of how new isolated patients are handled?*), how isolation precautions are communicated in their unit (*How do you know what isolation status a certain patient has / which precautions to use?*), and any suggestions they had to improve the current system (*How do you feel about the current isolation precaution signs? Would you have any recommendations to improve?*). Interviews were documented with detailed notes. Direct, unstructured observations were conducted in a representative sample of hospital settings (intensive care, emergency, general ward) with the purpose of observing multiple types of users interacting with the existing signage. Observations were documented through field notes and photos.

Following initial observations and interviews, *personas* representing distinct user groups were established that collectively describe the potential user population [17]. The design team established personas based on user patterns identified during interviews and observations. These personas were used by the design team and workshop participants to keep all potential users of the signage in mind and make sure that design alternatives were meeting the goals and needs of specific users. Further, a list of established design requirements was established as an evolving document that was refined throughout the design lifecycle with ongoing data collection. Throughout this process, both functional and non-functional design requirements were identified. Functional requirements described what the system should do in order to serve its purpose, whereas non-functional requirements concerned the physical, social, environmental, and technical constraints placed on the system design and its development [16].

### Designing alternatives – design thinking workshops

We conducted design thinking workshops with frontline HCPs based on an adapted methodology from the Stanford University Institute of Design including modes of empathising, defining, ideating, and prototyping [18]. Participants, grouped into pairs, were first prompted to interview their partners about the current challenges surrounding communication of isolation precautions (empathising) and then to synthesise information and insights from the interview into “problem statements” that described meaningful user challenges (defining). Based on these problem statements, participants then completed several ideation activities to generate new ideas and solutions – exchanging ideas and sharing feedback with their partner after each step (ideating). As a final step, participants used provided materials to create a prototype of their solution for communicating isolation precautions (prototyping). Workshop activities were documented with photos, detailed notes, and collected worksheets. Workshop documentation was then included in a qualitative analysis whereby all data was reviewed and grouped into thematic categories.

### Prototyping and evaluation

Building on the established list of design requirements, design ideas, and initial prototypes generated during the previous design phases, a process of iterative prototyping and user-testing began. Low-fidelity prototypes were produced using paper and pencil sketches, while higher-fidelity prototypes were produced using graphics software [19–21]. This process consisted of multiple cycles of user evaluation with continuous feedback from HCPs to make modifications to the prototype, which was then re-evaluated.

During the prototyping phase, symbols representing each isolation category (contact, droplet, and isolation), were also developed to be included in the overall isolation signage system. Multiple variants of each symbol were evaluated through *symbol judgement tests* and *comprehensibility tests* using paper-based surveys according to the methodology proposed by ISO 9186 [22]. During *symbol judgement tests*, participants were successively shown several variants of a symbol and its intended meaning. Participants were asked to estimate the percentage (from 0 to 100%) of HCPs and visitors to the hospital that could be expected to understand the meaning of each variant. The order with which variants of each symbol were presented was randomised for each survey to avoid order bias. The variants of each symbol with highest average score during judgement testing, meaning those that participants judged as being most likely to be understood, were then assessed through *comprehensibility testing*, during which a new group of participants were presented with a symbol and an image demonstrating the context in which they might find it. Participants were then asked to write exactly what they think the symbol means and what actions they should take in response to the symbol. Each participant saw one variant of each symbol and six different test versions were created with the order and combination of symbols randomised. The variant of each symbol with the highest percentage of correct responses was deemed the most comprehensible and was retained for further use during iterative prototyping and evaluation of the overall isolation signage.

The final signage prototypes underwent observations and expert review with infection prevention experts prior to being finalised by a professional graphic designer.

## Results

### Establishing design requirements – results of interviews and observations

**Fig. 2:**
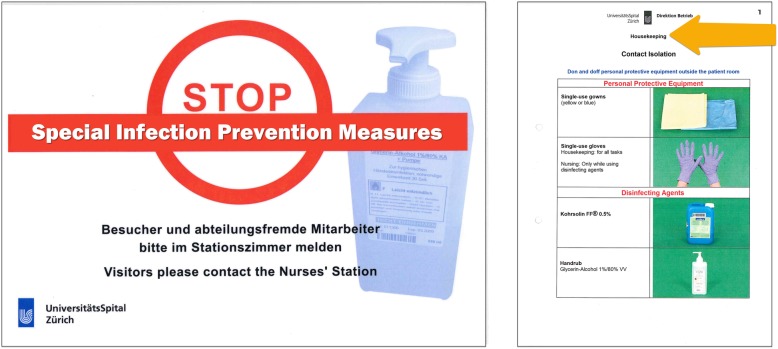
Existing isolation signage intended for healthcare providers for all isolation categories (left) and for housekeeping personnel specific to contact isolation (right). Legend: These signs have been translated from German to English for publication

The existing isolation signage system in our hospital was composed of a single poster for HCPs indicating only that “special precautions” were necessary and that visitors should contact the nurse’s station (Fig. 2). Additional signs existed for housekeeping personnel, which indicated the category of isolation, which protective measures were applicable, and which disinfecting agents should be used (Fig. 2).

Observations and interviews revealed several barriers to use of the existing isolation signage system. These barriers are presented in the following sections.

#### Confusion about target audience

When asked where they located information regarding isolation precaution measures, several HCPs erroneously referred to signage intended for housekeeping personnel. This was likely due to the inconspicuous or missing indication regarding for whom the signs were intended. Further, existing signage for HCPs simply indicated, “STOP, special infection prevention precautions” without indicating the type of isolation or which specific precautions apply, whereas signage for housekeeping personnel indicated specific precautions intended for cleaning procedures according to the isolation type. HCPs searching for specific guidance were frequently observed mistaking housekeeping measures, e.g. wearing gloves to protect skin from abrasive detergents, as intended for them.

#### Saliency

The existing signage for HCPs was placed next to or directly on the door of patients in single rooms together with an isolation cart with all necessary protective materials (e.g. gloves, gowns, masks). The isolation signage was often one of many posted signs and HCPs reported that the colours were not salient relative to other signage, meaning that the signs frequently went unnoticed. On intensive care units with patient bays rather than single rooms, signage was attached to isolation carts positioned near to the patient space. When the isolation signage was attached to the isolation cart, HCPs reported that it did not provide a salient visual cue because of the low signal-to-noise ratio. Interviewed HCPs shared that this resulted in them unknowingly entering into to spaces of isolated patients.

#### Trade-off between simplicity and complexity

**Fig. 3:**
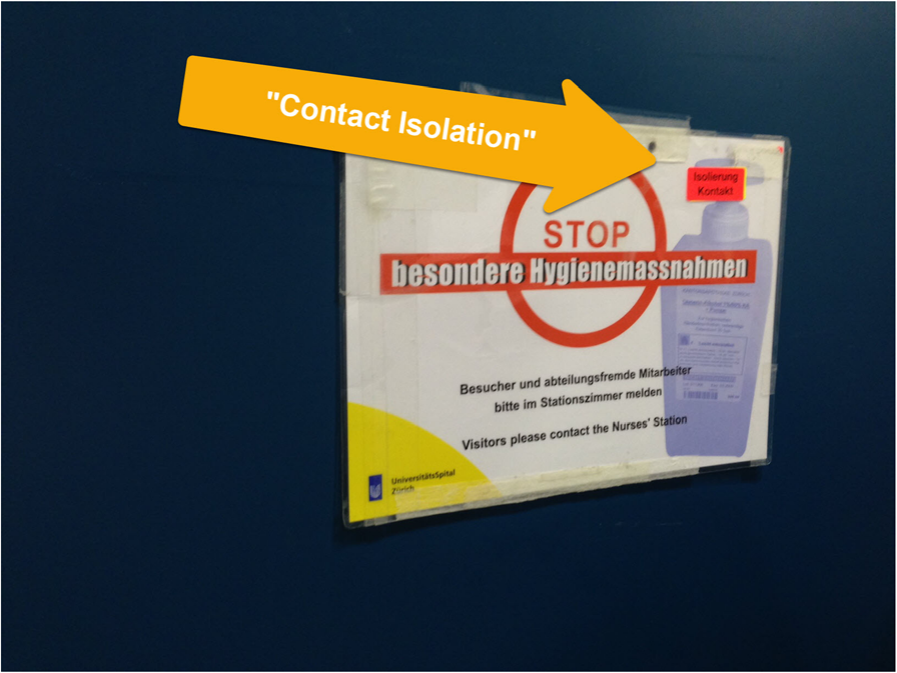
Existing signage for healthcare providers with “patch” to indicate category of isolation. Legend: This “patch” introduced by healthcare providers demonstrates that the existing signage was not offering sufficient information and that healthcare providers wanted the signage to indicate the category of isolation. The patch, indicated with an arrow, reads, “contact isolation”

**Fig. 4:**
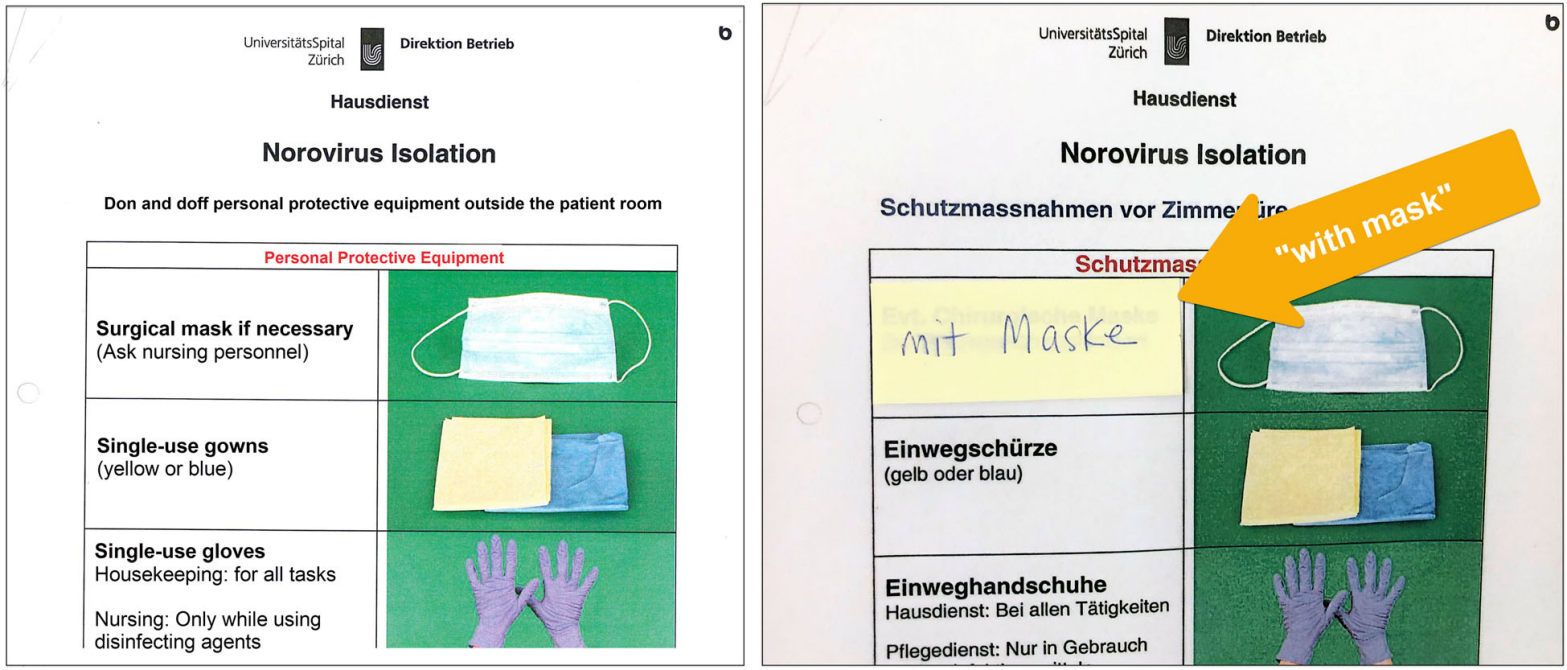
Existing signage for housekeeping (left) with “patch” to clarify ambiguous mask indication (right). Legend: This “patch” introduced by housekeeping personnel suggests that the existing signage provided ambiguous instructions and that they preferred a more simple instruction to don a mask. The patch, indicated with an arrow, reads, “with mask” instead of “surgical mask if necessary”. The existing signage (left) was translated from German to English for publication

The existing signage for HCPs neither indicated the category of isolation nor the necessary precautions to be taken. Observations of these signs in use revealed that HCPs desired more point-of-care information and they accordingly developed “patches” whereby they added stickers or wrote directly on the existing signage to add missing information, such as the category of isolation (Fig. 3). Alternatively, a simplifying “patch” had also been applied to the detailed housekeeping signs. Whereas the original sign indicated that a surgical mask “may be necessary” and that this should be discussed with nursing personnel, the patch saying “with mask” removed ambiguity and explicitly indicated the need to don a mask when cleaning the rooms of patients with norovirus (Fig. 4).

#### Method ambiguity

While some HCPs were able to cite the personal protective equipment (i.e. mask, gown) necessary for each category of isolation, many were unsure about the order with which these should be donned and doffed and when hand hygiene should be performed when entering and exiting the patient room.

#### Personas

Following interviews and observations, five personas were established to embody the identified goals and needs of key users of the signage system and to guide the rest of the design process (Table 1). These personas were printed and referred to throughout the subsequent study phases to keep a user-centred focus and to remind the design team and participating HCPs about the range of user needs to be considered.

**Table 1:** Established personas

Persona	Description	User requirements
Julia, Internal Medicine Nurse, Age 25	• Julia began working in the internal medicine ward at the USZ directly after finishing her nursing degree. Having freshly finished her schooling, she looks up to the more experienced nurses. • She is especially vigilant in her infection control practices, including isolation precautions, as this was a major focus of her training. • When she is not sure about which isolation precautions she should use for a patient, she (*erroneously*) references the sign on the housekeeping cart.	• Signage should offer multiple levels of information for frequent versus rare users. For example, recognition of the standardised signage colour may be enough for frequent users to recall the necessary precautions, whereas rare users need to be reminded of the specific precautions. • Target audience must be clearly distinguishable.
Sarah, Nursing assistant, Age 48	• Sarah has worked in several wards of the USZ. One of the highlights of her job is that it allows her to have a close relationship with patients. • She has a hard time bringing up isolation with her patients because she sympathises with the emotional effects this may have. For the same reason, she sometimes neglects to respect hygiene measures such as hand hygiene and wearing gowns, as she feels this puts a separation between herself and the patient.	• Signage should aim to establish social norms, increase acceptability of performing infection prevention measures.
Paul, Emergency Ward Physician, Age 42	• During medical school in Germany, Paul chose to specialise in emergency medicine because Dr. House was his favourite TV drama and he enjoys the challenge and rush to resolve critical situations. • Paul’s career took off quickly after he successfully published a highly cited paper in New England Journal of Medicine and he became known as an expert in fluid management in poly-trauma patients. • In the rush of acute care situations, infection control measures sometimes take a back seat, but no one dares to correct this senior physician.	• Signage must quickly communicate essential information and must not require extra time. • Barriers to performing isolation precautions (e.g. missing materials) should be removed. • Signage should aim to support psychological safety (e.g. speaking up).
Omar, Porter, Age 55	• Prior to moving to Switzerland with his family five years ago, Omar was an elementary school teacher in Tunisia. He came to Switzerland with no prior German knowledge but was able to begin working in the hospital while simultaneously taking German classes. • When he began working, he relied on clear photos and symbols to help him interpret written protocols.	• Signage must be able to communicate effectively with staff for whom German is not a native language, for example with self-explanatory images.
Teresa, Housekeeping staff, Age 46	• Teresa is specially assigned to work in the emergency ward, where they have specific cleaning and maintenance procedures from the rest of the hospital. Her native language is Portuguese, but her outgoing personality helped her to quickly learn German through chatting with her colleagues when she began working at the USZ, even picking up a Swiss German accent. • She has been working in the emergency ward for 12 years now, and although she has no medical training, she is excellent at what she does.	• Signage content may need to be adapted for specific settings. • Signage must be able to communicate effectively with staff for whom German is not a native language, for example with self-explanatory images.

Legend: Personas, fictional characters based on input from real users, were established to understand the needs and goals of the individuals who will interact with the signage system and to guide the subsequent design process.

#### List of design requirements

The information gathered during interviews and observations were used to establish a list of functional and non-functional design requirements (Table 2). Additional design requirements were subsequently added to this evolving document throughout the design process. The *functional requirements* primarily concerned information that the signage should provide to different categories of personnel and visitors are alerted and informed about the necessary isolation precautions. The *non-functional requirements* concerned the aspects of the signage that are required to make it compatible with the overall environment to which it will be introduced as well as non-functional requirements on the system level that needed to be addressed for the signage system to function optimally. One such requirement is that the signage must be consistent with corporate design guidelines at the USZ, which indicate the colours and fonts that should be used for internal and external communication.

**Table 2:** Identified design requirements

Functional requirements (what the system should do)	
Information dissemination	• Draw the attention of anyone entering the room that special precautions must be taken. • Inform any person entering the patient room what isolation status that patient has. • Inform any person entering the patient room about the isolation precautions they must employ according to the patient’s isolation status. ▪ Inform *healthcare personnel* about required personal protective equipment. ▪ Inform *cleaning staff* about required protective equipment, adapted disinfectants, and prioritised cleaning measures. ▪ Inform *visitors* about required personal protective equipment, or instruct them to see a staff member. • Inform any person entering the room about the order in which precautions (e.g. donning and doffing personal protective equipment, hand hygiene) should be performed. • Inform anyone transporting the patient about the transmission-based precautions that should be respected. • In addition to the three main categories of transmission-based precautions (contact, droplet, and airborne), signage should also be designed to communicate combined or “light” isolation precautions specific to the USZ.
USZ guidelines	• Be consistent with USZ guidelines for isolation precautions. The three main isolation categories include: ▪ Contact isolation: don gown ▪ Droplet isolation: don surgical mask ▪ Airborne isolation: don FFP2 mask • Communicate combined isolation precautions: ▪ Contact + Droplet isolation: don gown and surgical mask ▪ Contact + Airborne isolation: don gown and FFP2 mask
Non-functional requirements (constraints on the system and its development)	
Maintenance restraints	• Must be easy to hang and remove on an as-need basis. • If printed on paper, must either be laminate so that it may be cleaned and reused, or single use.
Accessibility and confidentiality requirements	• Should include graphics and symbols to accommodate non-native German speaking personnel. • Signage should avoid potential patient stigmatisation and should not disclose any confidential patient information. • Signage must be accessible to individuals with colour vision deficiency, for example by avoiding problematic colour combinations and employing both colours and symbols to convey messages over multiple channels.
Physical environment requirements	• Signage must be noticeable relative to other signs in the healthcare setting (appropriate single-to-noise ratio). • Some indication of the patient’s isolation status should travel with the patient at all times so that the appropriate transmission-based precautions can be communicated at all times (e.g. when the patient is outside of the room where the sign is posted) – this could be attached to the patient herself or to the patient’s bed. • Signage for housekeeping personnel should be of a portable size (A4 or smaller) so that it can fit on the cleaning trolleys.
Organisational environment requirements	• The signage should be able to quickly communicate the required actions, so that healthcare providers do not need to slow down (‘break the rhythm’) to interpret them. • If sign will be paper-based, it should be easily accessible to staff on the ward when an isolated patient arrives (e.g. printed copies from intranet) or purchasable. • Any posted signs must be consistent with the *Corporate Design Guidelines.* • Information communicated through signage must be consistent with evidence based-guidelines, such as the CDC 2007 guidance, as well as institutional infection control guidelines.
Technical environment requirements	• Colour printer must be available if units are to print their own copies of paper-based signage. • Intranet connection must be available if units are to download the signs. • Staff must be able to post the signage either by using an adhesive material, or the signs may be printed directly onto an adhesive sheet. • Handling of signage material should be cost-effective and practical in every-day use. • It should be possible to update the signage system easily in case new hospital rules/guidelines are introduced.

Legend: Design requirements identified during interviews and observations served as a guide to ensure that subsequent designs met user needs while satisfying functional and non-functional requirements. USZ, University Hospital Zurich; CDC, Centers for Disease Control.

### Designing alternatives – design thinking workshops

Two ideation workshops were conducted. Several recurring themes were identified, as discussed in the following sections.

#### Forcing function

**Fig. 5:**
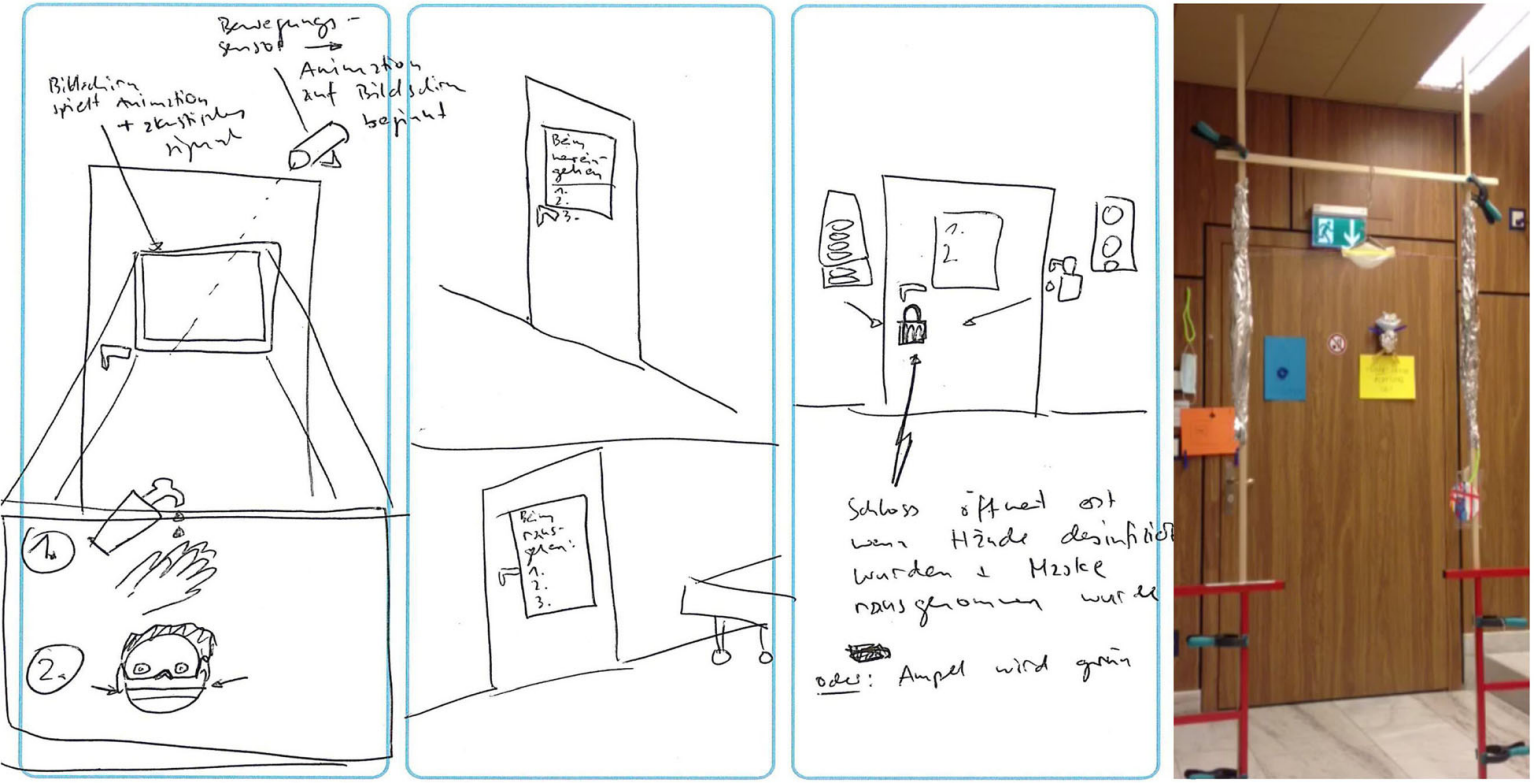
Workshop example of “forcing function” during ideation (left) and prototyping (right) phases. Legend: This extract from a design thinking workshop shows one participant’s proposal to use a “forcing function” to ensure that hands are disinfected prior to entry. The middle shows a door handle that only opens when alcohol is detected. The right shows a prototype featuring the forcing function of a mask suspended in the doorway, through which clinicians must walk and thereby don masks upon room entry

Several workshop participants presented ideas that employ the engineering concept of forcing function, a behaviour-shaping constraint, in order to ensure compliance with isolation precautions. During one workshop, participants working on the problem statement, “how might we ensure that required isolation measures are respected 100% of the time,” ideated around forcing function solutions to detect if appropriate personal protective equipment had been donned prior to opening the patient door (Fig. 5). Due to safety and feasibility concerns with this idea, during the prototyping phase these participants instead employed masks suspended in the doorway through which healthcare providers must walk, thereby automatically donning a mask upon room entry. Such solutions must be assessed to ensure their safety prior to introduction. Employing such forcing functions would address issues of forgetting, which participants confirmed is one of the main reasons for procedural noncompliance.

#### Not “breaking the flow of work”

**Fig. 6:**
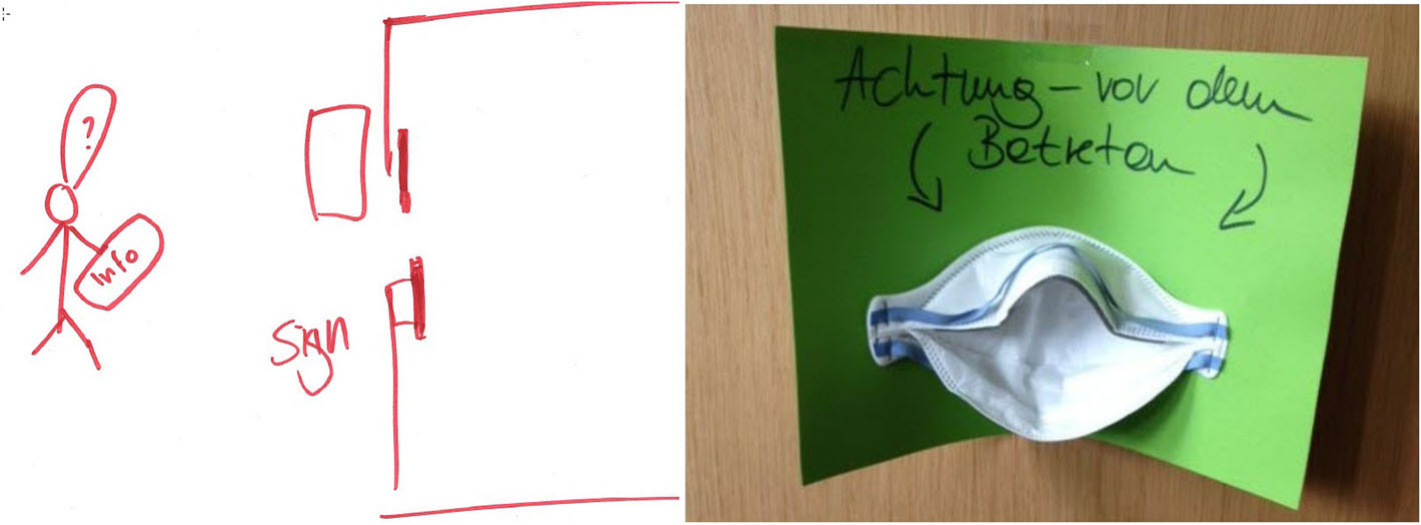
Idea (left) and Prototype (right) of signage from design thinking workshop. Legend: This prototype, developed during a design thinking workshop, sends a strong visual cue about the necessary precautions. The sign reads, “Warning, before entering …”

Several participants expressed that a major barrier to compliance with isolation precautions is the time necessary to find out which precautions are applicable and locate and don the materials necessary for compliance. This was expressed in the problem statement, “how might we save time while respecting isolation precautions?” Participants proposed multiple solutions. Indicating on the signage both the type of isolation as well as the necessary precautions eliminates the cognitive load required for those entering the room and saves time necessary to identify the relevant guidelines and materials. Figure 6 demonstrates both the ideation and prototyping of one such solution, which sends a strong visual cue indicating the type of protective equipment that should be employed.

#### Placement of visual cues

Another theme identified during the workshops was that the current provision of visual cues (only at the entrance to the patient room) is inadequate. When prompted about where visual cues should be located, the majority of participants indicated that some cues should also be located inside the patient room. The most commonly proposed locations for visual cues included: at the entrance to the patient room, at the foot of the patient bed, and next to patient charts.

### Prototyping and evaluation

#### Symbol development and judgement test

During design thinking workshops, participants generated ideas and sketches about how to portray the three main isolation categories (contact, droplet, and airborne) as symbols (Additional file 1). Variants of each isolation symbol [*contact isolation* (*n* = 4), *droplet isolation* (*n* = 5), and *airborne isolation* (*n* = 6)] were then designed employing the same themes that emerged from the participants themselves, in order to be consistent with user mental models and thus increase comprehension. Respondents’ judgements of how many of their colleagues would understand the symbols ranged from 17 to 78% (Additional file 2). Of note, the variants that pictured two whole human figures performed better in judgement testing than those without the human figure or part of the human body (e.g. only hands). The highest rated variants of each symbol were retained and further examined through a comprehensibility test.

#### Symbol comprehensibility test

**Fig. 7:**
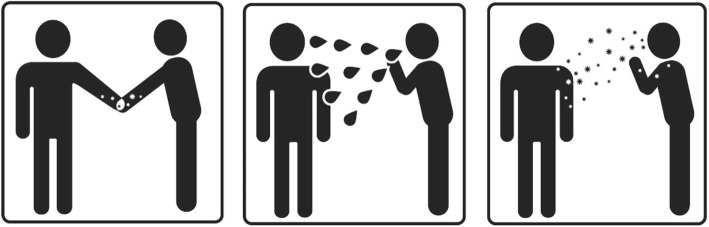
Symbols for contact (left), droplet (middle) and airborne (right) isolation. Legend: The variants of each symbol with the highest judgement and comprehensibility ratings were retained for use in further signage development phases

Detailed results of the comprehensibility testing can be found in Additional file 2. Whereas the symbols for contact and droplet isolation were correctly understood by a majority of participants, the symbols for airborne isolation were sometimes incorrectly interpreted as meaning droplet isolation. The three variants with the highest comprehensibility and least conflicting interpretations were retained for use in further signage prototypes (Fig. 7).

### Iterative prototyping and evaluation

The process of iterative prototyping and evaluation of isolation signage occurred in parallel to the testing of symbols. During this process, several prototypes emerged with varying levels of sophistication. In the earliest stages, ideas were expressed on paper in the form of sketches. Those sketches with the most positive feedback from the target population were then transformed into more sophisticated prototypes. As prototypes were continually evaluated through informal discussions with frontline HCPs, their feedback provided insights that led to further refining of consecutive prototypes. Experts in infection prevention and control were regularly consulted to ensure accuracy of the signage content. This iterative design process, as documented in Additional file 3, continued until saturation was achieved (i.e. until no new feedback was given).

### Final signage solution

**Fig. 8:**
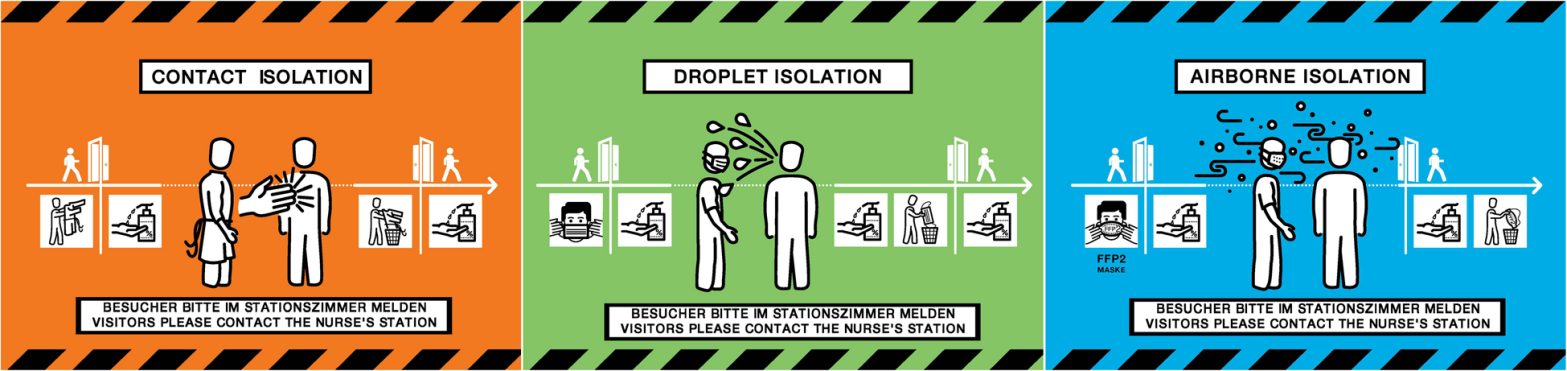
The final signage solution for contact (left), droplet (middle) and airborne (right) isolation. Legend: The final solution, to be printed with one sign per A4 sheet, incorporates several features to satisfy the identified design requirements. These signs have been translated from German to English for publication

**Fig. 9:**
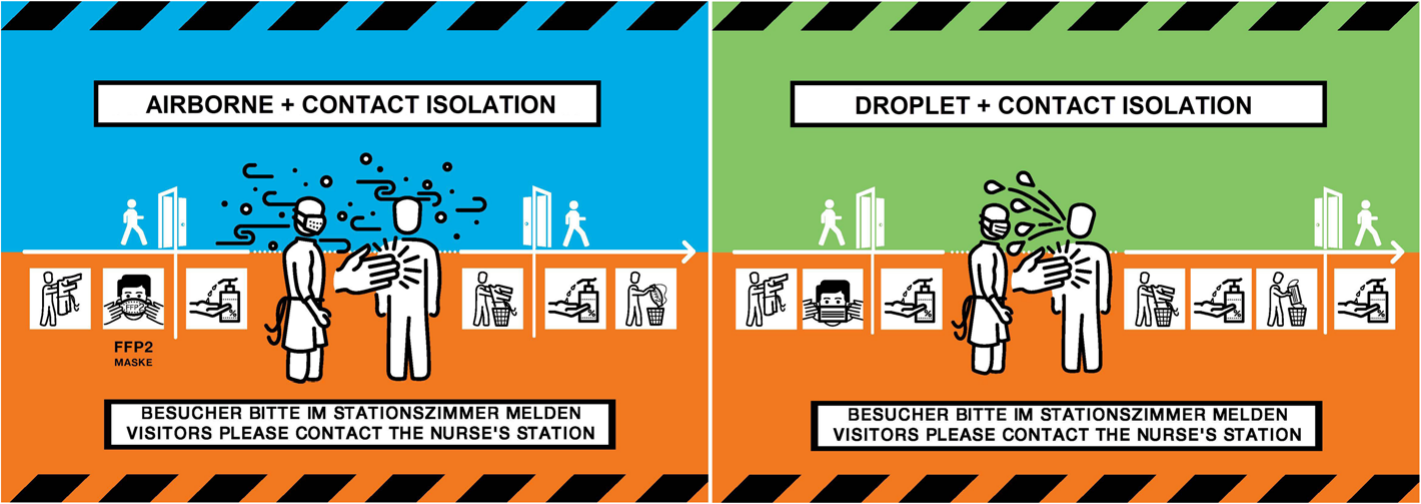
The final signage solution for combined precautions**.** Legend: These signs have been translated from German to English for publication

The final solutions that resulted from the iterative prototyping and evaluation process were shared with a graphic designer to produce the final signage system for the three main isolation categories (Fig. 8) as well as two signs for combined precautions (Fig. 9). This solution features a caution tape graphic and salient colours to draw attention to those entering the room that special precautions must be taken. The isolation categories are communicated dually through the colour, which is standardised throughout all isolation precaution documents, and the prominent symbol portraying the transmission pathway. The specific precautions are portrayed in the order in which they must be performed, removing method ambiguity. The use of text is limited and the signage relies largely on user-friendly graphics to support comprehension regardless of language proficiency. The signage is available for download on the intranet and can be printed with one sign per A4 sheet of paper. Beyond the scope of the physical signage, hospital guidelines have also been introduced that limit the amount and type of information that can be posted on patient doors, which increases the saliency of remaining signage. In collaboration with the infection prevention department, the housekeeping department has also revised their isolation precaution guidelines and brochures to eliminate confusion with signage intended for HCPs.

The signs were implemented by e-mailing heads of wards to inform them of the new signage, announcement and posting of the new signs on the intranet server, and physically distributing printed signs to each hospital ward according to number of beds. Additional copies were made available for order. Physical copies and links to the previous signage were removed.

## Discussion

This paper describes the successful application of a user-centred, participatory design process to develop a signage system for communicating isolation precautions. The resulting signage system aims to serve as a visual cue for HCPs, remove ambiguity, and elicit safe infection prevention behaviours to ultimately prevent the transmission of infectious agents.

Our analysis of existing isolation signage revealed that ambiguity was indeed a major factor responsible for non-compliance with transmission-based precautions in our hospital. This is consistent with the findings of Gurses et al., who propose systems ambiguity as a framework to explain procedural non-compliance in healthcare [8]. Ambiguity in our institution was identified mainly in the form of task ambiguity (not knowing which guidelines are applicable for which patients) and method ambiguity (not knowing how or in which order to complete a particular guideline). This was due to the lack of indication about which category of isolation a patient was under or which preventative measures were applicable. To specifically address barriers related to task and method ambiguity, the new signage was designed with the intent of bringing isolation guidelines directly to the frontline, where their use is indicated, also referred to as placing “knowledge in the world” [23, 24].

The symbols and colour-scheme developed to portray isolation categories (contact, droplet and airborne) were deemed to be an important part of the overall signage with the intention that these may become standardised throughout the institution and thus increase recognition and compliance. The comprehensibility evaluation of these symbols revealed that the airborne and droplet isolation symbols were the most often confounded. This is not surprising due to the similar means by which both airborne particles and droplets can be propelled into the environment through coughing and sneezing. In order to further distinguish these symbols, they have been associated in the new signage with standardised colour scheme, where airborne is blue, droplet is green, and contact is orange. The standardisation of these colour schemes is intended to further aid in the distinction of different isolation categories and make them quickly recognisable once they have been learned through repeated exposure.

It is commonly accepted that “concept-related” symbols, which are abstract representations of the referent or subject they represent, require more training before they can be understood by viewers [25]. However, by designing symbols for isolation precaution signage such that they are consistent with mental models, this project aimed to achieve a design that requires minimal training and that may also be intuitively understood. Interestingly, comprehensibility testing revealed that symbols showing the whole human figure were rated higher than those without the human figure or with an abstract part of the human body (e.g. only hands). This finding is consistent with results of the Hablamos Juntos Report, who evaluated the usability of several symbols for use in way-finding signage in hospital settings, and found that symbols with the whole human body were best understood [12].

In addition to comprehensibility benefits, including the human figure, particularly human eyes, may be important as it relates to emotional design. Multiple studies have examined the effect of images of human eyes on cooperative behaviour, such as an “honour system” method of payment to an honesty box to pay for drinks in a coffee room [26]. Such studies have found that the presence of eye images act as a subtle cue and that people paid as much as three times more often for their drinks when human eyes were displayed, as opposed to when a neutral control image was displayed [27]. Studies in infection prevention settings, however, have had mixed success in reproducing this effect [28–30]. Further studies on this topic are warranted to better understand how signage may be employed to prime cooperative behaviour and thus improve guideline adherence.

The field of infection prevention and patient safety is rife with opportunities to actively include HCPs in user-centred design processes to improve healthcare environments and practises. The participatory design approach was well suited for this inquiry as it allowed us to actively involve stakeholders with essential insights, thereby establishing ownership and increasing chances of acceptance and sustainability of the new signage. The benefits of collaborating with frontline HCPs extend beyond the scope of the resulting design, to also improve the image of the Infection Control department as a resource and to nurture the possibility of further collaboration. Recent reviews on the use of participatory approaches such as experience-based co-design or co-production, in which patients and healthcare staff are engaged to improve health services, report outcomes related to the value of patient and staff involvement, the quantity and quality of ideas and suggestions to modify practices, and tangible changes in service delivery and user experience [31, 32].

Some limitations of this study should be considered. During this study, we were faced with the challenge of changing institutional guidelines for isolation precautions. This prevented a quantitative analysis of change in practice and presented a challenge during the evaluation with frontline staff, who were not yet familiar with the new guidelines and thus had a tendency to focus on the discrepancies between the “new” vs. the current guidelines, rather than signage design. The latter was addressed through discussion with participants about the possibility of new evidence-based practices and also by presenting frontline users with prototypes that were consistent with their existing mental models, i.e. consistent with current guidelines. Also, the introduction of such signage is intended to serve as a visual cue and to remove ambiguity regarding which precautions should be used and when. The introduction of such signage, however, does not entirely remove other known barriers to compliance with isolation precautions, such as “lack of time” [33] or “availability of protective clothing” [5]. It is thus imperative that the introduction of such signage takes place within the context of a systems approach, ensuring, for example, that the necessary time and material resources are also available in order to mitigate these barriers.

## Conclusions

In conclusion, this study resulted in a user-centred signage design solution and set of comprehensible symbols to remove ambiguity and to make compliance with evidence-based guidelines easy and intuitive for HCPs and visitors to the USZ. The introduction of signage, particularly for infection control, offers a promising opportunity to make guidelines available directly at the frontline, where their use is indicated, thereby reducing the need for training and standard operating procedures. This article further describes the application of a user-centred participatory design process, which has great potential for application in the field of hospital infection control to design solutions that make performing infection prevention behaviours easy and intuitive.

## Supplementary information

**Additional file 1.** Pictorial portrayal of isolation categories

**Additional file 2.** Results of Judgement and Comprehension testing

**Additional file 3.** Iterations of Isolation Signage System

## Data Availability

The datasets used and/or analysed during the current study are available from the corresponding author on reasonable request.

## Abbreviations

CDC: Centers for Disease Control
HAI: Healthcare-associated infections
HCP: Healthcare provider
ID: Infectious diseases
IPC: Infection prevention and control
USZ: University Hospital Zurich
