# Supplementary material for: User-centred participatory design of visual cues for isolation precautions
Source: Antimicrob Resist Infect Control. 2019 Nov 19;8:179. doi: 10.1186/s13756-019-0629-9 (PMC6862753; doi:10.1186/s13756-019-0629-9)

**Additional file 1: Pictorial portrayal of isolation categories**

**Contact Isolation**

When asked to portray “contact” isolation, almost all participants drew hands – the vehicle by which physical contact most commonly occurs. Several participants drew two hands, demonstrating that physical contact was occurring. Red was a commonly used colour.


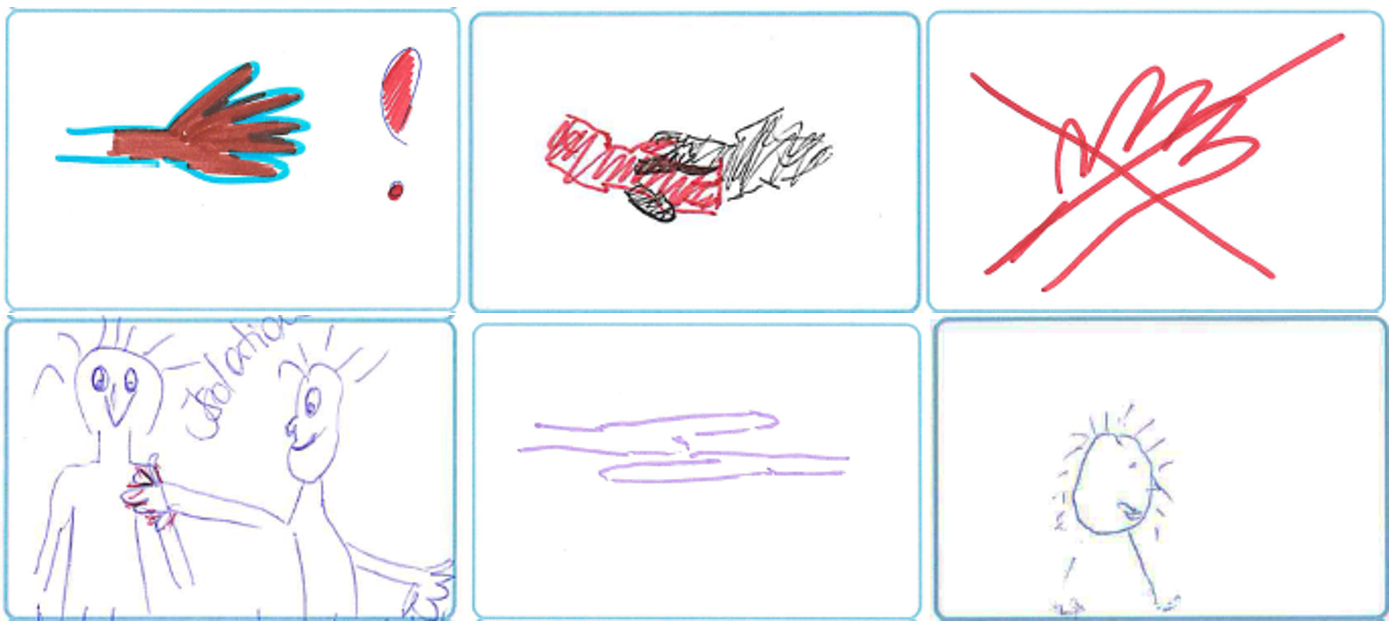


**Droplet isolation**

Almost all participants portrayed droplet isolation using the shape of a drop of water. Several also portray movement from the source of the droplets, e.g. through coughing or sneezing. One participant drew a bug inside of a droplet, making visible the typically invisible pathogens that travel through droplets. Blue was the primary colour used. The drawing shown on the bottom far right portrays a pathogen using a trampoline, limited by the space it can jump, just as pathogens are limited by their projection in droplets when coughing or sneezing.


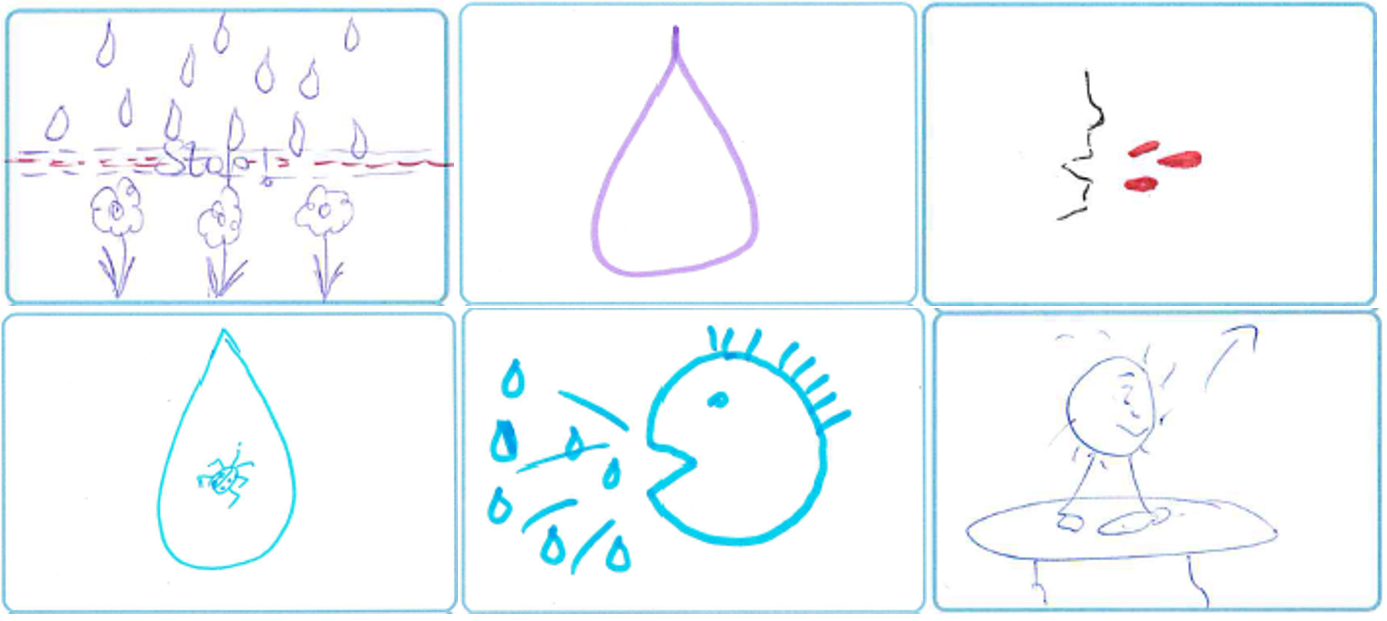


**Airborne isolation**

Several images emerged to represent airborne isolation. These included clouds, wind, and non-enclosed particles. Particles in 2 pictograms are “free” to move about in the space, not held within a cloud or other shape. Such is the nature of airborne transmission, particles that become aerosolized are not contained. The drawing on the bottom right, again portraying the mode of airborne transmission, shows a pathogen with a jet pack – able to travel long distances, not confined by contact or limited by where it falls.


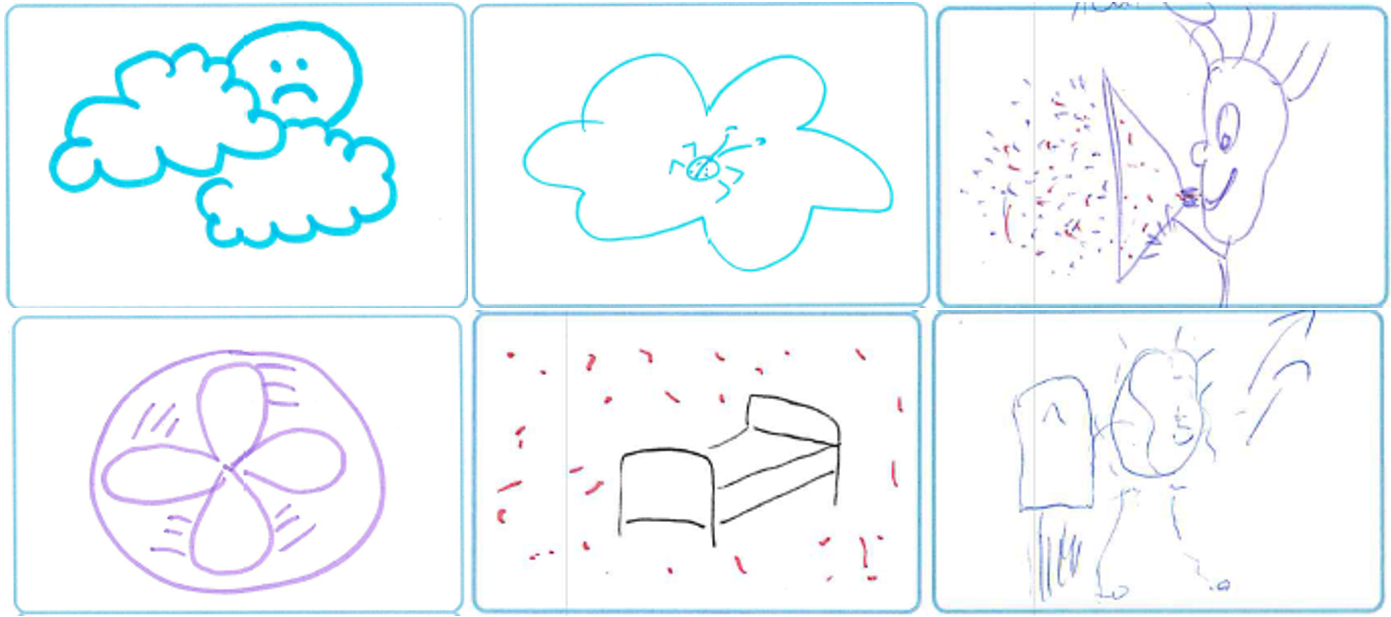

Supplement: Supplementary file 1 — Additional file 1. Pictorial portrayal of isolation categories [file 13756_2019_629_MOESM1_ESM.docx]
