# Supplementary material for: User-centred participatory design of visual cues for isolation precautions
Source: Antimicrob Resist Infect Control. 2019 Nov 19;8:179. doi: 10.1186/s13756-019-0629-9 (PMC6862753; doi:10.1186/s13756-019-0629-9)

# Annex 2: Results of Judgement and Comprehension testing

*Symbol judgement testing*

A total of 15 symbols were assessed by 10 respondents during the judgement test. Respondents included male (n=2) and female (n=8) nurses (n=6), physicians (n=2), and lab technicians (n=2), with ages of 31-50 (n=6) and over 50 (n=4). The symbols assessed included variants of three referents: *contact isolation* (n=4), *droplet isolation* (n=5), and *airborne isolation* (n=6), pictured above. Respondents’ judgements of how many of their colleagues would understand the symbols ranged from 17-78%. The highest rated symbols for each category were retained and further examined through a comprehensibility test.


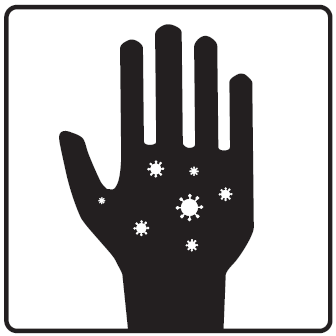

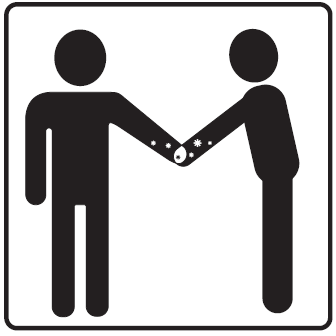

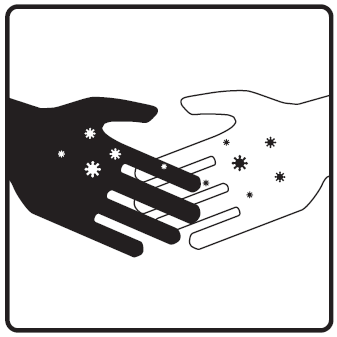

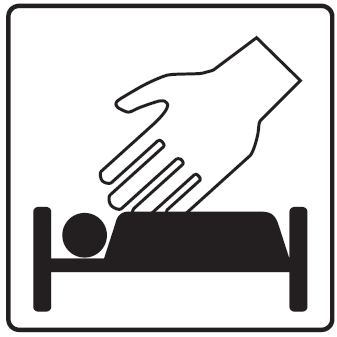

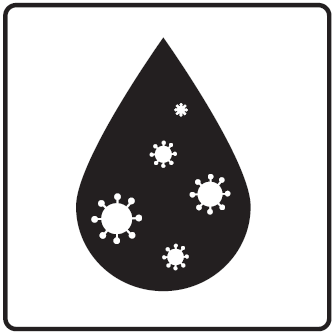

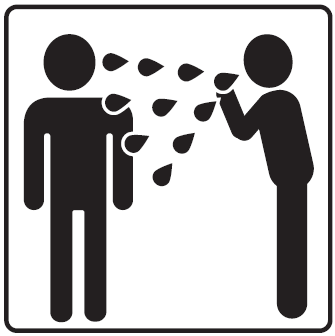

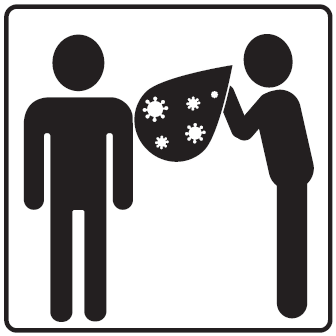

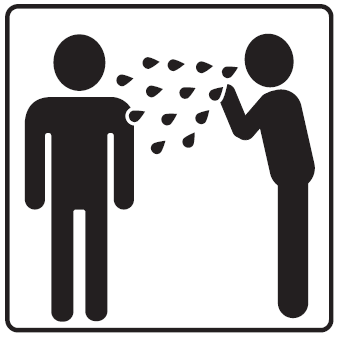

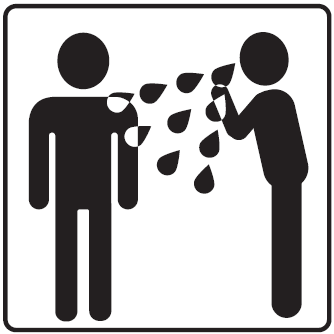

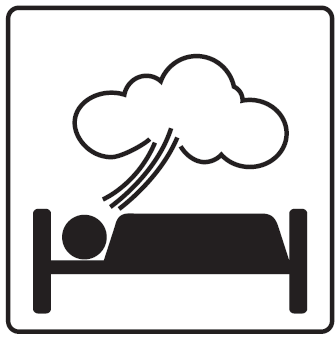

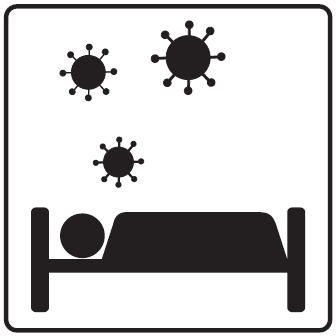

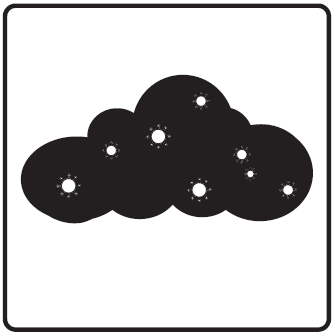

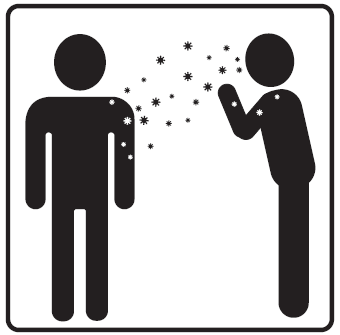

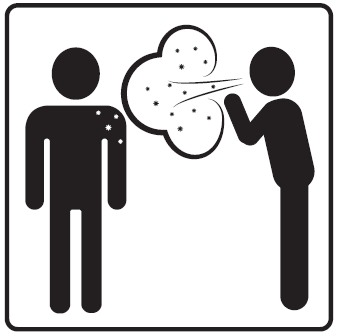

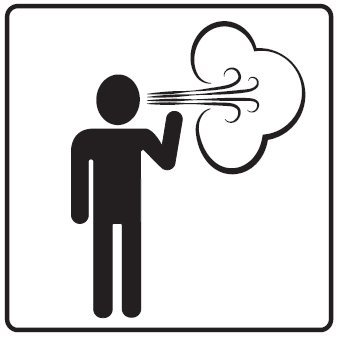


Percentages represent respondents’ judgements of how many healthcare personnel and visitors they expect would understand the given symbols.

*Symbol comprehensibility testing*


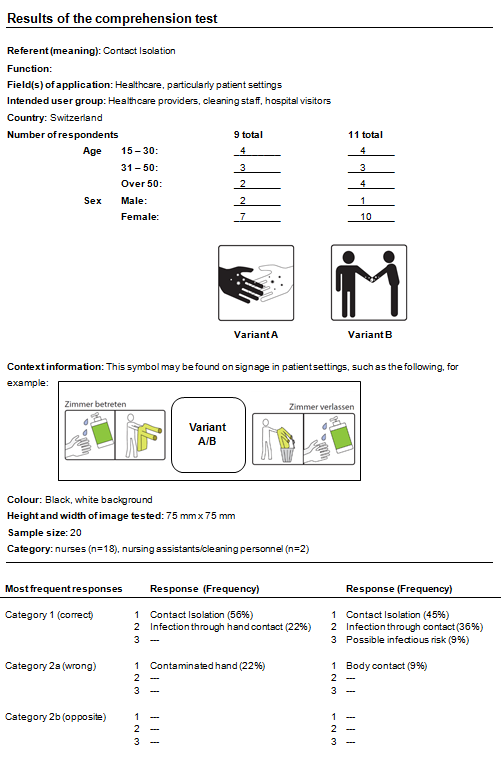

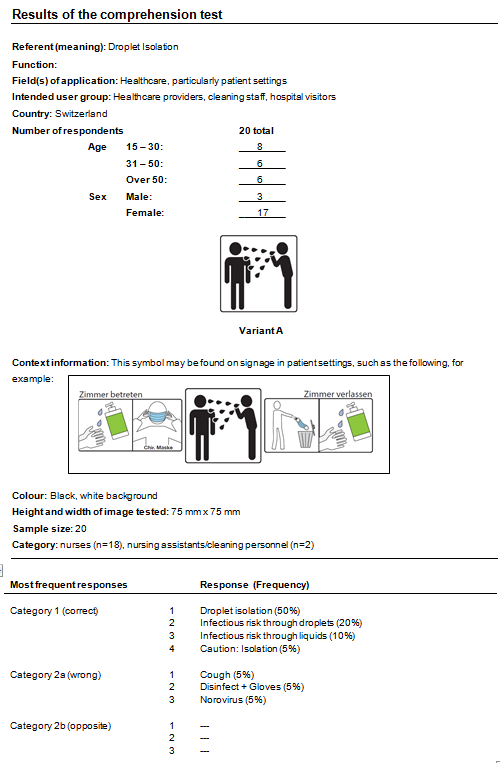

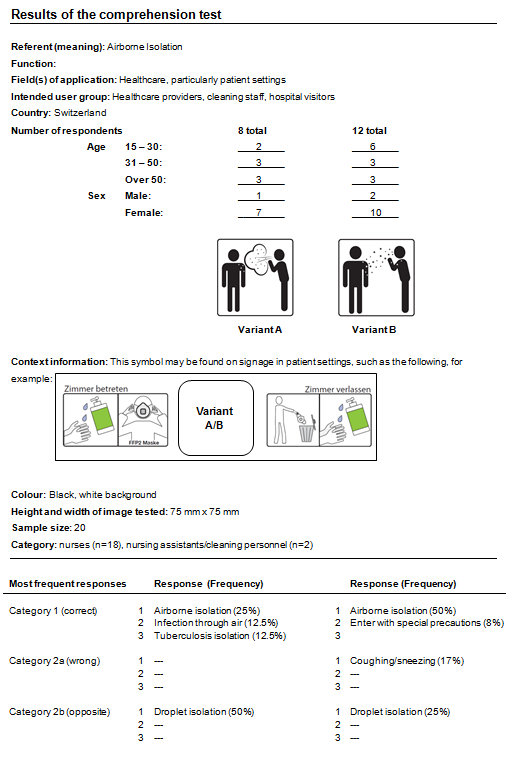

Supplement: Supplementary file 2 — Additional file 2. Results of Judgement and Comprehension testing [file 13756_2019_629_MOESM2_ESM.docx]
