# Supplementary material for: User-centred participatory design of visual cues for isolation precautions
Source: Antimicrob Resist Infect Control. 2019 Nov 19;8:179. doi: 10.1186/s13756-019-0629-9 (PMC6862753; doi:10.1186/s13756-019-0629-9)

Annex 3: Iterations of Isolation Signage System

# Iteration 1

*Description -* Each sign in this first iteration is composed of three elements: 1) a title indicating the category of isolation, 2) an illustration portraying the mode of pathogen transmission, 3) illustrations of the necessary protective measures.

*Feedback -* too playful, illustrations of pathogen transmission are redundant, focus should be on the protective measures (e.g. donning of gowns and masks).


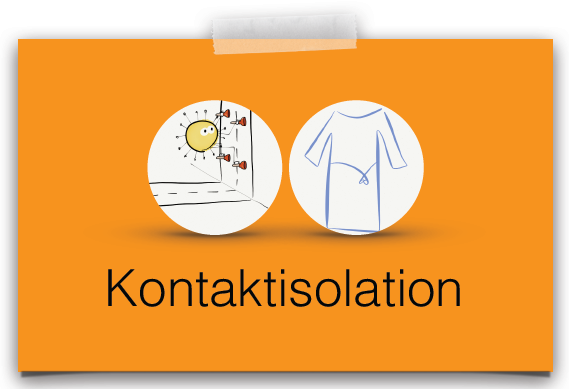

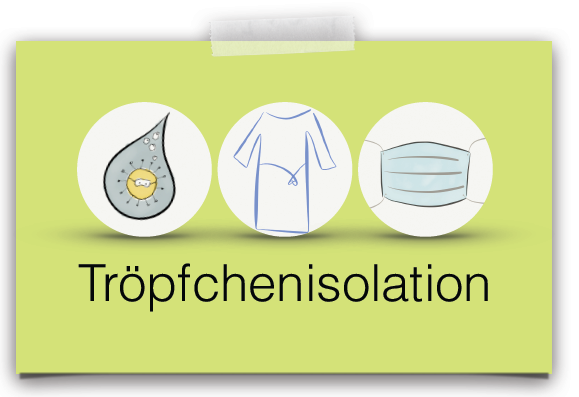

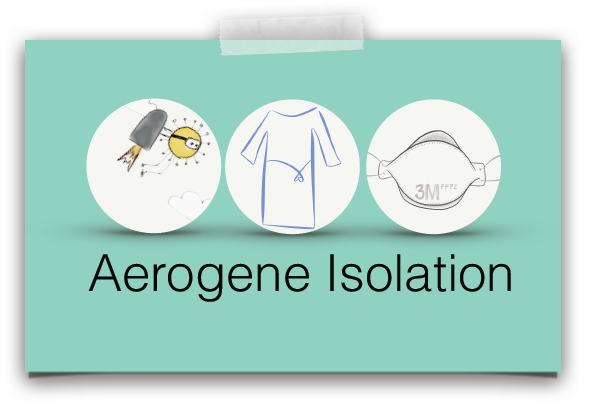


# Iteration 2

*Description -* This iteration was designed to emphasize the necessary protective measures to be respected upon room entry.

*Feedback* – illustrations still too playful, visually unattractive


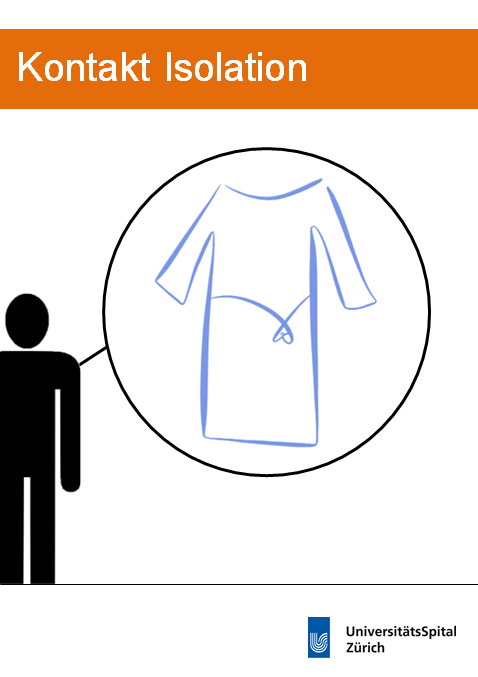

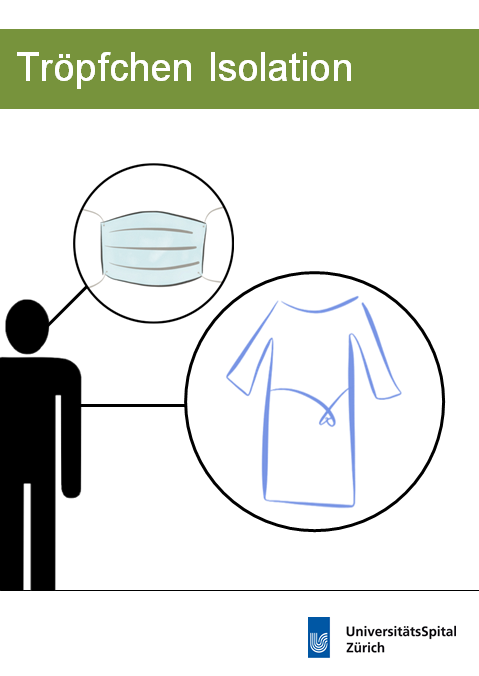

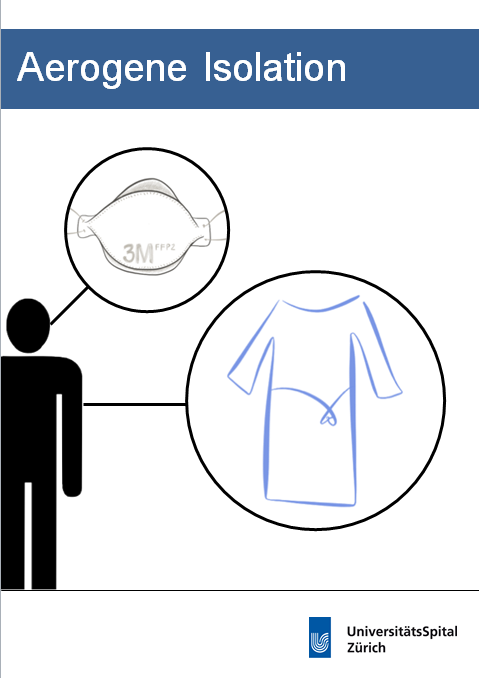


# Iteration 3

*Description –* This iteration integrated suggestions from the infection prevention team that we could use isolation precaution signage to also emphasize the important of standard precautions, hand hygiene and donning gloves in case of potential exposure to body fluids.

*Feedback* – standard precaution glove symbol was misunderstood as indication to don gloves for all isolated patients


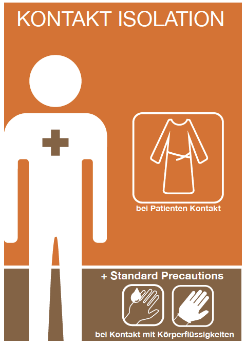

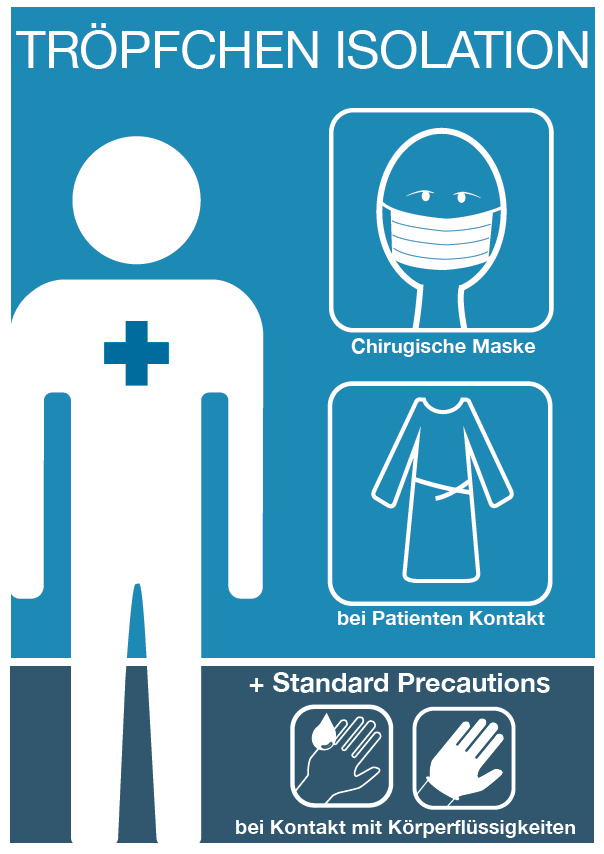

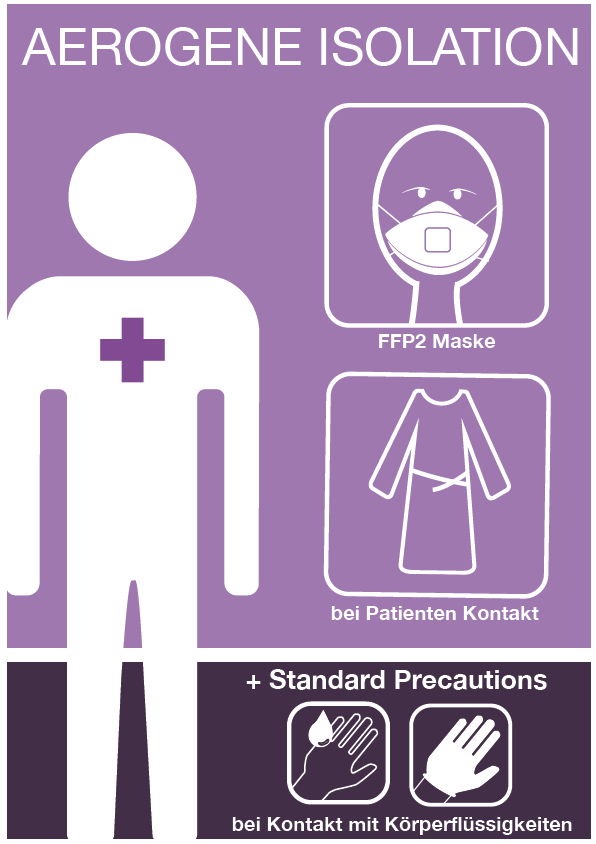


**Iteration 4 (pre-final)**

*Description –* This iteration integrated an important standard precaution by integrating hand disinfection indications into the donning and doffing process. The category of isolation (contact, droplet, or airborne) is clearly indicated at the top of each poster. The yellow and black stripe draws healthcare providers’ attention to the patient’s special isolation status. The isolation symbols are prominently positioned to emphasize the mode of transmission.


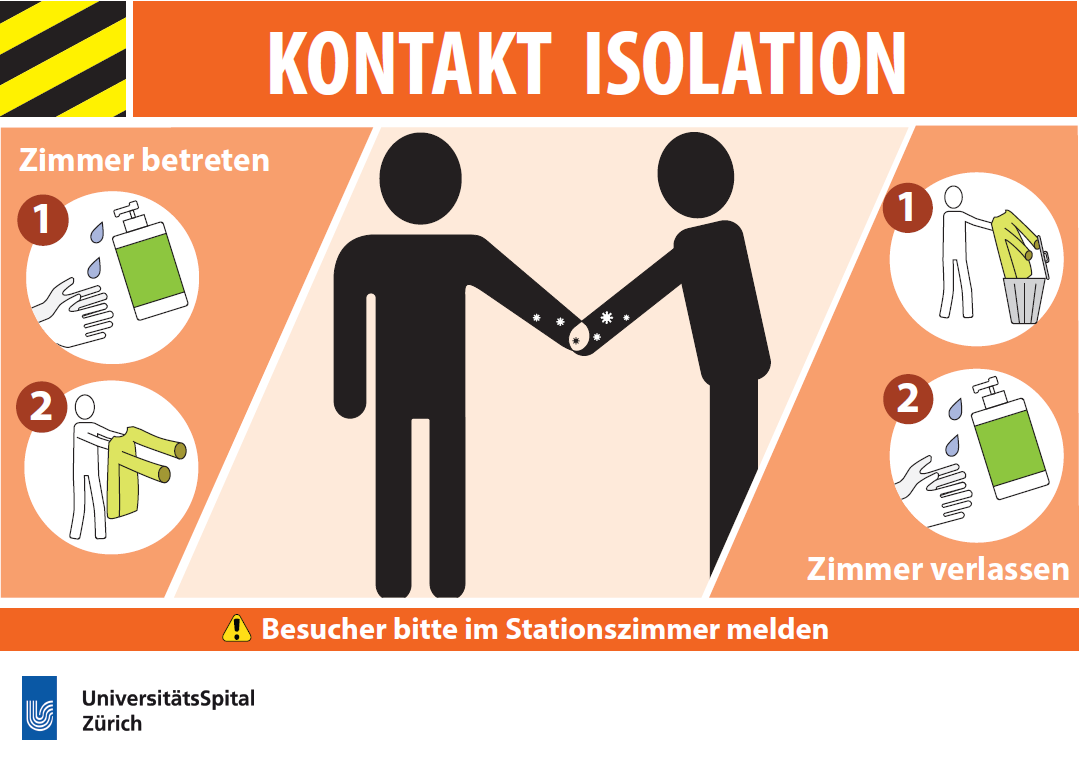


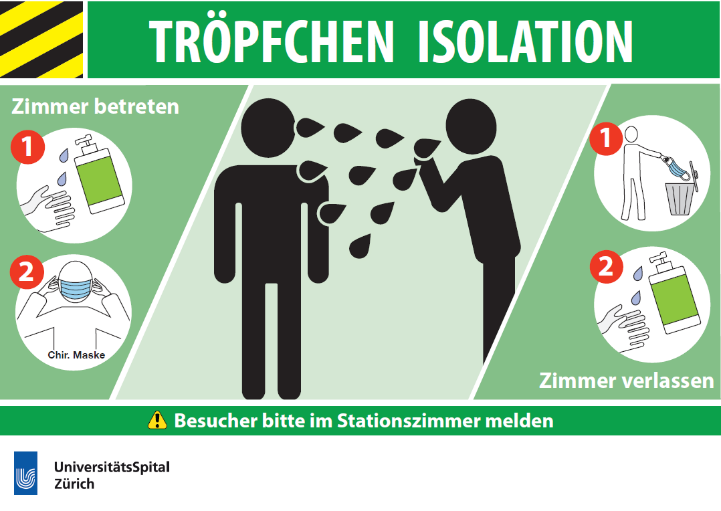


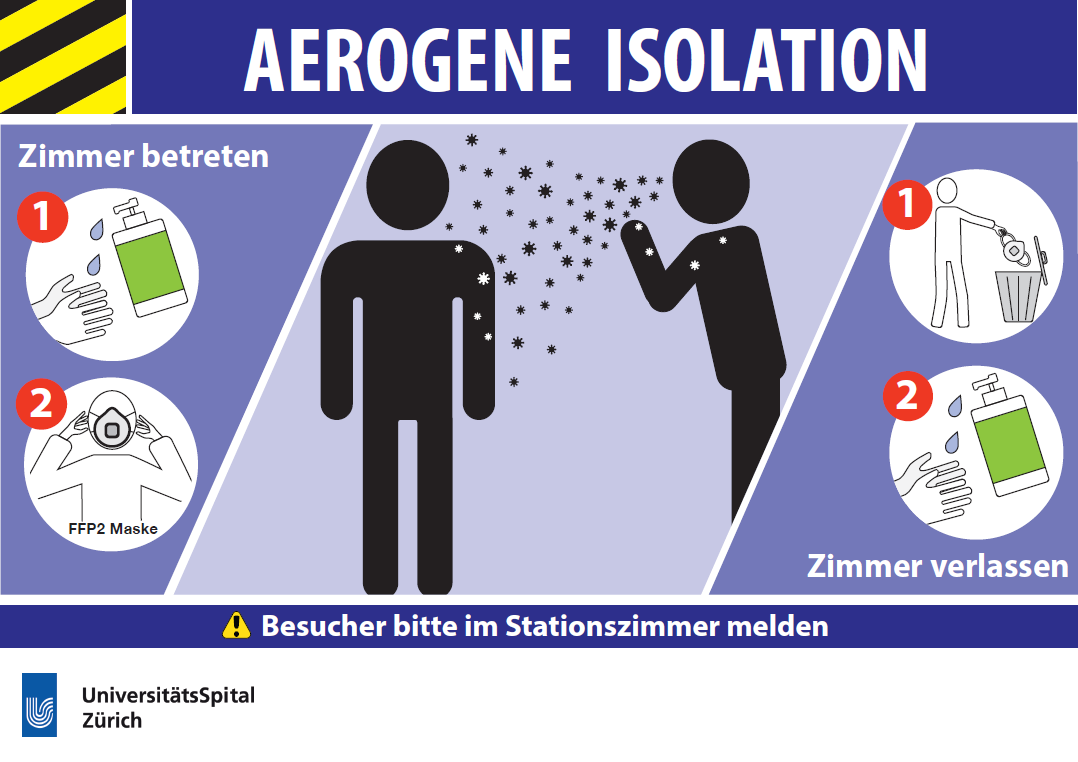

Supplement: Supplementary file 3 — Additional file 3. Iterations of Isolation Signage System [file 13756_2019_629_MOESM3_ESM.docx]
